# A missed opportunity: underutilization of inpatient behavioral health services to reduce injection drug use sequelae in Florida

**DOI:** 10.1186/s13011-021-00383-w

**Published:** 2021-05-31

**Authors:** Austin E. Coye, Mackenzie T. Jones, Kasha J. Bornstein, Hansel E. Tookes, Joan E. St. Onge

**Affiliations:** 1 University of Miami Miller School of Medicine, 1600 NW 10th Ave #1140, Miami, FL 33136 USA; 2 Department Medicine, Division of Infectious Diseases, University of Miami Miller School of Medicine, Miami, FL 33136 USA; 3 Department of Medicine, Division of General Internal Medicine, University of Miami Miller School of Medicine, Miami, FL 33136 USA

**Keywords:** People who inject drugs, Opioids, MOUD, Inpatient addiction services, Addiction

## Abstract

**Background:**

People who inject drugs (PWID) suffer high morbidity and mortality from injection related infections (IRI). The inpatient setting is an ideal opportunity to treat underlying substance use disorder (SUD), but it is unclear how often this occurs.

**Objectives:**

To quantify the utilization of behavioral health services for PWID during inpatient admissions for IRI.

**Methods:**

Data for all hospital admissions in Florida in FY2017 were obtained from the Agency for Healthcare Administration. Hospitalization for IRI were obtained using a validated ICD-10 algorithm and treatment for substance use disorder was quantified using ICD-10-Procedure Coding System (ICD-10-PCS) codes.

**Result:**

Among the 20,001 IRI admissions, there were 230 patients who received behavioral health services as defined by ICD-10-PCS SAT codes for treatment for SUD.

**Conclusions:**

In a state with a large number of IRI, only a very small portion of admissions received behavioral health services. Increased efforts should be directed to studying referral patterns among physicians and other providers caring for this population and increasing utilization of behavioral health services.

## Background

People who inject drugs (PWID) are a vulnerable patient population who often require recurrent hospitalization for sequelae of drug use [1–4]. This vulnerable population experiences increased mortality risk after hospitalization as well as high risk for readmission [5]. A national 2016 analysis found approximately 24% of patients hospitalized for opioid overdose had at least one readmission within 90 days of discharge and 3% were readmitted for overdose [6]. The high rehospitalization rates in this population signify a need for more effective medical care during hospital stays and provide critical windows of opportunity for hospital physicians to engage these patients in treatment for underlying substance use disorder(s) (SUD).

Significant recent evidence demonstrates myriad benefits of initiating treatment for underlying SUD in inpatient settings [7–12]. Peterson et al. found that identifying patients with SUD while in-hospital is an effective method to target prevention services that reduce opioid-related morbidity and mortality [12]. Wakeman et al. demonstrated that implementation of an inpatient addiction consult team resulted in decreased admissions for patients with SUD [8]. Kimmel et al. determined treatment with medication for opioid use disorder (MOUD) following hospitalization for injection drug use-associated infective endocarditis reduced mortality during the month that MOUD was received [7]. Despite the evidence that initiation of treatment for SUD during hospital admissions reduces morbidity and mortality in this population, previous literature indicates that these services are likely underutilized [13, 14]. However, the extent to which addiction services are utilized in inpatient settings is not well known.

The aim of this large-scale study was to assess utilization of addiction services during the hospitalization of 20,001 PWID admitted for injection related infection (IRI) across every Florida hospital during Fiscal Year 2017 (FY2017).

## Methods

We conducted a retrospective review of patients hospitalized for IRI in Florida during FY2017, using the Agency for Health Care Administration (AHCA) Hospital Inpatient Limited Data Set. AHCA disclaims responsibility for analysis, interpretations, and conclusions. PWID admissions for IRI were identified using an algorithm combining International Classification of Diseases, 10th Revision (ICD-10) codes indicating drug use (opioids, cocaine, amphetamine, overdose, other psychoactive); and common infectious sequelae (endocarditis, osteomyelitis, bacteremia-and/or-sepsis, and skin-and-soft-tissue infections (SSTI)). The algorithm was validated using British Columbia Hepatitis Testers Cohort data and noted to have a sensitivity of 63% and a specificity of 100% [15].

PWID admissions between 18 and 75 years of age and with length of stay (LOS) < 60 days were included. Patients who expired during hospitalization were excluded. Age and LOS restrictions were included to increase specificity of the PWID identification algorithm and patients that expired during the stay were excluded because they may have been considered too sick to receive inpatient treatment of SUD [16].

ICD-10-Procedure Coding System codes were used to assess treatment for SUD. These are labelled in ICD-10 as codes for substance abuse treatment, however we will refer to them as Substance Addiction Treatment (SAT) to avoid the use of stigmatizing language as outlined by the National Institute on Drug Abuse [17]. Codes for SAT (PCS SAT) included detoxification services, individual counseling, group counseling, medication management, and pharmacotherapy. Individual and group counseling modalities included cognitive, behavioral, cognitive-behavioral, 12-step, interpersonal, vocational, psychoeducational, motivational enhancement, confrontational, continuing care, spiritual and pre/post-test infectious disease. Individual psychotherapy included cognitive, behavioral, cognitive-behavioral, 12-step, interpersonal, interactive, psychoeducation, motivational enhancement, confrontational, supportive, psychoanalysis, psychodynamic, and psychophysiological. Medication management and pharmacotherapy for SAT comprised nicotine replacement, methadone, levo-alpha-acetylmethadol, disulfiram, naltrexone, naloxone, clonidine, bupropion, psychiatric medication, and nicotine replacement medications.

## Results

This analysis includes 20,001 PWID admitted to Florida hospitals during FY2017 for IRI. Admissions most often included bacteremia-and/or-sepsis (53%) followed by SSTI (48%). Opioids were the most common drug recorded (52%). The majority (55%) of patients had publicly subsidized insurance, while 33% were uninsured. Median LOS was nine days (IQR: 3–12). (Appendix).

Among the 20,001 PWID admissions included, there were 230 patients who received behavioral health services as defined by PCS SAT, representing fewer than 2% of all PWID admissions. Among these admissions, female patients comprised 44%. Most (*n* = 200) were identified as non-Hispanic. The majority (*n* = 196) were identified as white. The most common service rendered was detoxification (*n* = 220), followed by Individual Psychotherapy for SAT (31 admissions). Only 29 of 20,001 admissions recorded medication management or pharmacotherapy for SAT (including methadone, naltrexone, and/or clonidine) during the study period (Table 1).

**Table 1:** Demographics of PWID Admissions who received ICD-10-PCS Substance Addiction Treatment

	Inpatient Admissions (%)
**Biological Sex**	
Male	128 (56)
Female	102 (44)
**Ethnicity**	
Hispanic or Latino	25 (11)
Non-Hispanic or Latino	200 (87)
Other	5 (2.0)
**Race**	
Black or African American	20 (8.7)
White	196 (85)
Other	14 (6.1)
**Age (years)**	
18–29	40 (17)
30–39	68 (30)
40–49	62 (27)
50–59	37 (16)
60–75	23 (10)
Mean Age (years)	40.7
**Insurance Status**	
Federal	47 (20)
State, County, Local	57 (25)
Uninsured	41 (18)
Private Insurance	83 (36)
Other	2 (0.9)
**Discharge Status**	
Discharge or transferred	193 (84)
Discharged to Hospice	2 (0.9)
Left AMA	35 (15)
**Hospital Service Utilization**	
Median Length of Stay	6 days
**Substance Addiction Treatment**^a^	Admissions (%)
Connection to any Addiction Services	230 (100)
Detoxification Services	220 (96)
Individual or Group Counseling	31 (13)
Medication Management or Pharmacotherapy	29 (13)

^a^Admissions could include more than one ICD-PCS Code

## Discussion

The data presented reveal that referral to addiction services and prescription of SAT medications for hospitalized PWID are underutilized in Florida. The median LOS was nine days among all PWID admissions, indicating ample time for SAT initiation. Data from this statewide sample demonstrates significant opportunities to increase utilization of these services, potentially reducing readmission rates, mitigating preventable toxicological and infectious morbidity, and reducing mortality. Previous studies demonstrate that PWID admissions come at great social and economic cost to Florida and nationwide [15, 18]. Limited billing for PCS-SAT modalities through analysis of billing and coding records in this statewide sample suggests major gaps exist during inpatient treatment of underlying SUD [15, 19].

Other studies suggest similar deficiencies in implementation of best practices for hospitalized PWID. A national survey of hospitalists indicated that while 84.5% reported “often or always” caring for patients with opioid use disorder (OUD), 88.9% “rarely or never” initiated buprenorphine in the inpatient setting [20]. A 2019 analysis of Veterans Health Administration data found only 16% of veterans began any SUD treatment following initial diagnosis [13]. Only 2.7% began MOUD within 14 days of their first encounter [13]. In a retrospective review of patients hospitalized with injection drug use-associated infective endocarditis between 2004 and 2014, Rosenthal et al. found fewer than 25% of PWID received psychiatry or addiction medicine consultation [14]. Addiction was mentioned in only 56% of discharge summary plans and only 7.8% of patients had planning for MOUD [14]. Retrospective analysis also found that over 25% of those patients were deceased by 2016 and the median age at death was 40.9 years [14]. While our findings are similar to others, this study uses a unique algorithm to quantify SAT in a large inpatient sample, one that can replicated easily in other states to monitor treatment and outcomes of initiatives to increase SAT.

A major limitation of this study is the structure of ICD-10 coding for drug use disorders and SAT. To date, the ICD-10 lacks specific code(s) for PWID, complicating efforts to study the care of this patient population and necessitating algorithmic approaches to identification of hospitalized PWID [15, 21, 22].While highly specific, the statistical algorithm used in this study underestimates the real number of hospitalized PWID in Florida during the study period. In addition to built-in stigmatizing language such as “abuse”, there is no specific category for major medications such as buprenorphine or acamprosate and it includes vague categories such as “psychoactive medication.” ICD-10-PCS codes for SAT pharmacotherapy include drugs such as nicotine replacement therapy and bupropion, which have no known utility in the medication-assisted treatment of opioid use. Additionally, levo-alpha-acetylmethadol is not used in the United States. Utilization of ICD-10-PCS codes risks possible lack of coding, miscoding, or underreporting of services rendered. Although this methodology does not capture all psychiatric services rendered to hospitalized PWID, it is consistent with other reports and suggests general underutilization deserving of further exploration.

While we did not capture brief counseling about SUD or other informal counseling during inpatient stay, brief counseling is still likely underutilized. One study observed that 22% of hospitalized patients seen by an addiction consultation-liaison service received only brief counseling without formal referral and without essential risk mitigation modalities [23]. Brief counseling is not as effective as other interventions. In a randomized trial comparing in-hospital MOUD initiation versus brief counseling, MOUD was associated with increased engagement in treatment and reduced illicit opioid use [24]. Patients may also have been referred to outpatient behavioral health treatment or to harm reduction organizations, neither of which was captured in this study.

One proposed explanation for the paucity of interventions offered to PWID may be presumed lack of social stability or resources for treatment adherence. However, a Swedish study found parity in 12-month retention rates among MOUD program participants with and without strict social stability requirements [25]. Another study found homelessness positively predicted MOUD initiation among hospitalized adults seen by an addiction consult service, indicating that hospitalization may be an important opportunity to engage typically harder to reach populations [26]. Insurance status was also likely not the primary barrier in receiving behavioral health services as only 36% of the admitted patients receiving services in that study had private insurance.

The latter years of the 2010s saw the first prolonged, multiyear decline in average life expectancy in the United States in over a century, secondary to complications of hazardous opioid use [27]. This mortality rate has amplified considerably in the setting of the COVID19 pandemic: early results indicate national Emergency Medical Services activations for opioid-related cardiac arrests in 2020 occurred 48.5% above baseline [28]. This dramatic worsening of preventable opioid morbidity and mortality emphasizes the critical imperative of adapting evidence-based best practices for addiction during hospitalization.

The American College of Physicians, the National Institutes of Health, and the Infectious Diseases Society of America have issued calls for action to implement and scale up effective SUD treatment in healthcare settings [29–31]. The attendees of a National Academies of Sciences, Engineering, and Medicine workshop on “Integrating Infectious Disease Considerations with Response to the Opioid Epidemic” proposed an action plan that includes screening for OUD in all relevant settings and immediate prescription of effective medication for OUD and/or opioid withdrawal symptoms [32]. In an article on the diagnosis and management of OUD in hospitalized patients, Herscher et al. emphasize the roles that hospitalists play in engaging patients with OUD in treatment [33]. Writing on the management of co-occurring OUD and infectious disease in inpatient settings, Eaton and Vettese echo the importance of initiating MOUD for patients experiencing infectious sequelae, noting that management of injection-related infections “is incomplete without addressing the underlying [SUD].” The authors recommend a number of initiatives focused on reducing stigma, addiction medicine consultation, improved pain management, and implementing harm reduction resources [34].

While the findings of the present study highlight the underutilization of services to address SUD in hospitalized patients, these guidelines and calls for action have the potential to enhance the role hospitalists play in the treatment of PWID. However, hospitalists cannot achieve these goals alone; other systemic and structural changes are necessary to improve high-quality SUD treatment access. The undersupply of addiction services likely contributes to low SUD service utilization. Involvement of inpatient psychiatry and addiction teams are effective means to reduce addiction severity and substance use, initiate inpatient care, and transition to outpatient care for SUD [10, 35]. A study of patients with SUD admitted for serious infection demonstrated addiction medicine consultation improved rates of SUD treatment, increased likelihood of completion of antimicrobial therapy, and reduced readmission rates [9]. However, a national survey of hospitalists found that only 67.9% of respondents reported access to addiction specialists. Those with access to addiction specialists were 4.4 times more likely to screen and three times more likely to refer patients for treatment than those without [20]. Unfortunately, the pipeline for physicians training in addiction medicine or addiction psychiatry is low-flow; during 2020–21 in Florida, only 13 physicians trained in accredited programs in these specialties [36]. Prioritizing expansion of these one-year training programs by including them in state and federal graduate medical education funding expansion could rapidly increase availability of addiction specialists. This lack of specialists also likely drives a paucity of outpatient services, presenting additional barriers for transitions of care of SUD is started inpatient [37, 38]. Education on SUD and MOUD has been a focus in Florida since 2019, with the call for and support of education programs at medical schools focusing on SUD, MOUD and opportunities for intervention [39]. Institutional establishment and/or partnerships with syringe access programs, expansion of provider education for OUD and elimination of procedural barriers including the “X-Waiver” and prior authorizations are other ways to reduce major treatment gaps [40].

## Conclusion

In a state with a large number of IRI, few PWID received behavioral health services, highlighting opportunities to address risk factors for increased morbidity and mortality. Enhanced funding for addiction specialists and harm reduction organizations, increased SUD education, and reduction of barriers to prescribing MOUD will support our hospital-based colleagues in their efforts to expand access to treatment in this population.

## Data Availability

The data that support the findings of this study are available from Agency for Health Care Administration but restrictions apply to the availability of these data, which were used under license for the current study, and so are not publicly available. Data are however available from the authors upon reasonable request and with permission of Agency for Health Care Administration.

## Appendix

**Table 2:** PWID Hospitalizations for Infections in Florida, FY 2017

Characteristic	PWID Admissions (%)
**Biological Sex**	
Male	10,779 (54)
Female	9222 (46)
**Ethnicity**	
Hispanic or Latinx	1608 (8)
Non-Hispanic or Latinx	17,930 (90)
Unknown	299 (2)
**Race**	
Black or African American	2281 (12)
White	16,694 (84)
Other	862 (4.4)
**Age (years)**	
< 29	3377 (17)
30–39	5071 (25)
40–49	3910 (20)
50–59	4219 (21)
60–75	3424 (17)
Mean Age (years)	44.5 (IQR 33–56)
**Insurance Status**	Admissions (%)
Federal	5625 (28)
State, County, Local	5045 (25)
Uninsured	6632 (33)
Private Insurance	2411 (12)
Other	124 (0.6)
**Length of Stay (Days)**	9.39 (IQR 3–12)
**Discharge Status**	
Discharge or transferred	15,913 (80)
Expired	755 (4)
Left AMA	3169 (16)
**Infection Type**^a^	
Skin and Soft Tissues	9586 (48)
Osteomyelitis	2669 (14)
Bacteremia/Sepsis	10,596 (53)
Endocarditis	1908 (10)

^a^Many admissions had more than one infection diagnosis coded per admission

## Abbreviations

PWID: People Who Inject Drugs
IRI: Injection Related Infections
SUD: Substance Use Disorder
ICD-10 PCS: International Classification of Diseases, 10th Revision, Procedure Coding System
SAT: Substance Addiction Treatment
PCS SAT: Procedure Coding System Codes for Substance Addiction Treatment
MOUD: Medication for Opioid Use Disorder
FY2017: Fiscal Year 2017
AHCA: Agency for Health Care Administration
SST: Skin and Soft Tissue Infection
LOS: Length of Stay
OUD: Opioid Use Disorder
